# miR-20a-5p/TGFBR2 Axis Affects Pro-inflammatory Macrophages and Aggravates Liver Fibrosis

**DOI:** 10.3389/fonc.2020.00107

**Published:** 2020-02-13

**Authors:** Xiutao Fu, Jingbo Qie, Qingchun Fu, Jiafeng Chen, Yinpeng Jin, Zhenbin Ding

**Affiliations:** ^1^Department of Liver Surgery and Transplantation, Liver Cancer Institute, Zhongshan Hospital, Fudan University, Shanghai, China; ^2^Minhang Hospital and Institutes of Biomedical Sciences, Shanghai Medical College, Fudan University, Shanghai, China; ^3^Shanghai Public Health Clinical Center, Fudan University, Shanghai, China

**Keywords:** miR-20a-5p, liver fibrosis, TGF-β signaling pathway, inflammation, TGFBR2

## Abstract

Combined inhibition of programmed death-ligand 1 (PD-L1) and transforming growth factor-β (TGF-β) displayed additive anti-tumor response in a subgroup of cancer patients, highlighting the importance of understanding the multifaceted roles of TGF-β in immunity and fibrosis. In the present research, we show that TGF-β signaling pathway, controlled by miR-20a-5p and transforming growth factor-β receptor 2 (TGFBR2), alters the inflammation and fibrosis processes in liver. We performed integrated analysis of differently expressed miRNA (DEM) associated with liver fibrosis and screened miR-20a-5p out as a key regulator in inflammation-driven liver fibrosis. We subsequently conducted Kyoto Encyclopedia of Genes and Genomes (KEGG) pathway enrichment analysis of the genes targeted by miR-20a-5p. And the result showed that 12 target genes were significantly enriched in TGF-β signaling pathway. Further study showed that miR-20a-5p was down-regulated and involved in inflammation during liver fibrosis in human and mouse samples, indicating that miR-20a-5p and inflammation are functionally linked during liver fibrosis progression. To uncover the underlying pro-inflammatory mechanism of miR-20a-5p in liver fibrosis, we selected and verified TGFBR2, which is a key functional receptor in TGF-β signaling pathway, as a direct target gene of miR-20a-5p. The downregulation of miR-20a-5p in liver fibrosis resulted in TGFBR2-activated TGF-β signaling pathway, followed by the activation of macrophage and extracellular matrix (ECM) production by hepatic stellate cell (HSC). Our results identify the miR-20a-5p/TGFBR2 axis as a key regulator of TGF-β signaling, and highlight the critical role of miR-20a-5p in the development of liver fibrosis.

## Introduction

Therapeutic antibodies against the programmed death-1 (PD-1)/programmed death-ligand 1 (PD-L1) axis has been approved to treat multiple tumors, but only not effective in all patients (1). It is well-known that transforming growth factor-β (TGF-β) is of importance in resistance to immune checkpoints inhibitors. Recently, M7824 (MSB0011359C), a bifunctional fusion therapeutic antibody against human PD-L1 fused to the extracellular domain of human transforming growth factor-β receptor 2 (TGFBR2) showed enhanced preclinical antitumor activity through simultaneously blocking the PD-L1 and TGF-β signaling pathways (2, 3). These results prompt us to understanding the multifaceted roles of TGF-β signaling pathway in immunity and fibrosis. Liver fibrosis is an essential pathological process that may deteriorate into liver cirrhosis and liver cancer, making it one of the leading causes for the high mortality and morbidity around the globe (4). Regardless of origins and etiologies, liver fibrosis developed from viral infection, alcohol, non-alcoholic steatohepatitis (NASH), and autoimmune diseases, featuring chronic, and pathological process (5). Relying on the studies of underlying liver injury, several evidences highlighted the important role of immune reactions (6). Liver cell damage tends to induce the secretion of pro-inflammatory factors, such as tumor necrosis factor-α (TNF-α), tumor necrosis factor-β (TNF-β), nuclear factor kappa-B (NF-κB), Interleukins (ILs), which sequentially stimulate the infiltration of inflammatory cells (7). Subsequently, excessive infiltration of inflammatory cells would render the liver more vulnerable to damage by preying upon liver cells and thus initiating fibrogenesis. An in-depth understanding about the underlying mechanism of liver fibrosis is the cornerstone to research the effective therapies for chronic liver diseases.

MicroRNAs (miRNAs) are endogenous, small non-coding RNA molecules that play essential part in various biological functions and numerous processes, such as immune response, cell proliferation, and apoptosis, through the post-transcriptional regulation of gene expression in cells (8). Increasing evidence indicated that aberrant expression of miRNAs are closely related to numerous types of cancer, as well as liver fibrosis (8–12). It's frequently reported that miRNA expression level in the serums or liver tissues of liver fibrosis patients is dominantly changed (13–15). Normally, miRNAs exacerbates liver fibrogenesis by incomplete matches with their host genes that are related to hepatic stellate cells (HSCs) activation, immune cell sensitization, as well as hepatocytes apoptosis (16, 17).

In our study, we demonstrated that the level of inflammatory cytokines in serum was upregulated in CCl_4_-treated mice, suggesting that inflammation is accompanied by liver fibrosis. Many previous studies reported that miRNAs drove liver fibrogenesis by regulating inflammation response. We performed integrated analysis of differently expressed miRNA (DEM) associated with liver fibrosis and screened miR-20a-5p out as a key regulator in inflammation-drove liver fibrosis. We subsequently conducted Kyoto Encyclopedia of Genes and Genomes (KEGG) pathway enrichment analysis of the genes targeting by miR-20a-5p. The result showed that 12 target genes were significantly enriched in TGF-β signaling pathway, which participated in the development of liver fibrosis. Further study indicated that miR-20a-5p was down-regulated and related to inflammation during liver fibrosis in human and mouse samples, indicating that miR-20a-5p and inflammation are functionally linked during liver fibrosis progression. To reveal the pro-inflammatory mechanism of miR-20a-5p in liver fibrosis, we selected and verified TGFBR2, a key functional receptor in TGF-β signaling pathway and a target gene of miR-20a-5p. The downregulation of miR-20a-5p in liver fibrosis resulted in TGFBR2-activated TGF-β signaling pathway, followed by the activation of macrophage and extracellular matrix (ECM) production by HSC. Our results highlight a critical function of miR-20a-5p in the development of liver fibrosis, and the reintroduction of miR-20a-5p provides a promising therapeutic strategy for clinical intervention of liver fibrosis.

## Materials and Methods

### Patients and Animal Model

Liver fibrosis specimens have been collected from 26 patients who were seeking treatment in our hospital and from 19 patients with liver diseases, except liver fibrosis. The published and well-acknowledged clinical guidelines were applied as the clinical diagnostic criteria for liver fibrosis. Written informed consent was obtained from the participants of this study and all participants were above 16 years old (Table S1).

CCl_4_-induced liver fibrosis mouse model was established by conducting intraperitoneal injection of carbon tetrachloride (CCl_4_; 0.6 mL/Kg body weight) in 8-week-old mice twice a week. The intraperitoneal injection lasted for 8 weeks. Male C57BL/6 mice were obtained from Shanghai SLAC Laboratory Animal Co., Ltd. All animals were treated humanely according to protocols approved by the Fudan University Committee on Animal Care and Use.

### Cell Lines and Cell Transfection

Immortalized mouse hepatocyte cell lines Hepa1-6 and macrophage cell line Raw264.7 were obtained from the Shanghai Institute of Biochemistry and Cell Biology, Chinese Academy of Sciences (Shanghai, China). Cells were grown in DMEM supplemented with 10% fetal bovine serum, 2 mM L-glutamine, and 100 units/ml penicillin/streptomycin. The miRNA mimics and negative control were transfected into Hepa1-6 cell line by using Lipofectamine™ 2000, in strict accordance with the manufacturer's instruction.

### Quantitative-PCR (qPCR) Analysis

Total RNAs were extracted from Hepa1-6 cell line and liver fibrosis specimens using Trizol (Invitrogen, CA, USA) and all total Nucleic Acid Isolation Kit (Ambion Inc., USA), following the manufacturer's instruction. miRNAs primers for reverse transcription were purchased from Huada Co. Ltd (Beijing, China). The experiment was performed three times using SYBR Premix Ex Taq (cat#RR420A, TaKaRa, Japan) to quantify the mean values of delta Ct and SD (standard deviation). miRNA expression level was normalized to the relative quantities of U6 to investigate fold change. The primers used for miRNA and mRNA quantification were listed in Table S2.

### FACS

Flow cytometry assay (using BD LSR Fortessa II) was carried out on hepatic non-parenchymal cells which are composed of the total profile of hepatic leukocyte population. The experiments were performed as published (18). The following pre-conjugated antibodies were used: CD11B (552850, BD bioscience), CD45 (553083, BD bioscience). Briefly, Hepatic macrophages were defined as viable CD45+ CD11B+ F4/80+ cells from digested livers and used to identify macrophage subsets. Subsets were expressed as proportions of total hepatic macrophages or CD45+ cells. And we collected 10,000 cells every time.

### Immunohistochemistry (IHC), Immunofluorescence (IF), and Western Blotting (WB)

Human and mouse liver tissues were processed for IHC, IF, and WB. Antibodies used in the present study are α-SMA (19245, CST), DESMIN (5332, CST), TGFBR2 (ab186838, abcam), p-Smad2 (18338T, CST), p-Smad3 (9520, CST), GAPDH (30201ES20; Yishen). Images were acquired using Olympus FV1000 confocal system with a 10X objective. The fluorescence was imaged using 552 nm/408 nm for mCherry /DAPI.

### ELISA

Mouse IL-6 (VAL604, R&D), TNF-α (VAL609, R&D), Mouse IL-18 (7625, R&D) ELISA kits were used following the directions of the manufacturer. Conditioned medium (100 μl) was collected from triplicate samples.

### Cell Viability Analysis

Cell viability was monitored using the Cell Counting Kit 8 (CCK8) method. Cells were inoculated onto a 96-well plate. Each well-contained 10,000 cells, and 6 repeats were used for every treatment. After 24 h, cellular proliferation was detected using a cell counting kit-8 (CCK-8, Yisheng). The effect on Hapa1-6 proliferation was evaluated by analyzing EC50 curves according to absorbance of cells (OD_450_).

### Determination of the Levels of miRNAs Related to Liver Fibrosis

The microarray file of liver miRNomes GSE40744 obtained from GEO database (http://www.ncbi.nlm.nih.gov/geo/) was referred to investigate miRNAs expression levels in our collected human fibrotic liver tissues and healthy controls. This miRNA microarray based on the platform of GPL14613 (Affymetrix microarray chip platforms) contained 18 fibrotic liver samples and 19 normal liver samples. GEO2R (19) is an interactive online tool and often used for gene expression analysis of microarray data through the GEO query and limma packages (20) available in R. The protocol was performed to investigate DEMs between normal, mild fibrotic, and advanced fibrotic liver samples. Adjusted *p* value of no >0.05 in combination with a |log_2_ (fold change) | of >1 were set as the threshold for the identification of DEMs.

### Prediction of Target Genes

The potential target genes of miR-20a-5p were analyzed by miRDB (21), TargetScan (22), and miRTarBase (23). The genes predicted by miRDB, TargetScan, and miRTarBase simultaneously were identified as the targets of DEM.

### Functional Enrichment Analysis and miRNA-gene Network Construction

The database that can be used for annotating, visualizing and integrated discovering of the predicted genes (DAVID 6.8, https://david.ncifcrf.gov/) was applied in performing the KEGG pathway enrichment analysis (24, 25). FDR of <0.05 was considered as statistically significant.

The target genes enriched in KEGG pathways were mapped to the STRING database (https://string-db.org/) to evaluation the intricate functional associations amongst target genes (26), and the miRNA-gene network was constructed and visualized by Cytoscape software (Version 3.6.0).

### Luciferase Activity Analysis

The partial sequences of TGFBR2 3′UTR which contained the wild or mutant binding sites of miR-20a-5p were amplified and then cloned into the pGL3-Basic luciferase vector (Promega, W.I.) with the aim of constructing pGl3-TGFBR2 (WT) and pGl3-TGFBR2 (Mut). Primers used in plasmid construction were as follows: forward 5′-CAGGCTGGGCCATGTCCAAA-3′ and reverse 5′-GTCAAATGCTAATGCTGRCATG-3′. The two plasmids were, respectively, co-transfected with miR-NC, miR-20a-5p mimic, anti-miR-NC, and anti-miR-20a-5p (Genomeditech). Forty eight hours later, the luciferase activity analysis was conducted on the Dual-Luciferase Reporter assay system (Promega, W.I.), in strict accordance with the instructions of the manufacturer.

### Statistical Analysis

The data of the present study were presented in the form of mean ± SD (standard deviation). Unpaired/paired Student's *t*-test was used to analyze the significance of miRNA diversity between the two groups. A *P* value of <0.05 (two-tailed) was set as a threshold to distinguish statistically significant difference. Linear regression was performed using Graphpad Prism 7 (GraphPad Software Lnc, USA).

## Results

### Inflammation Is Accompanied by Liver Fibrosis

**Figure 1:**
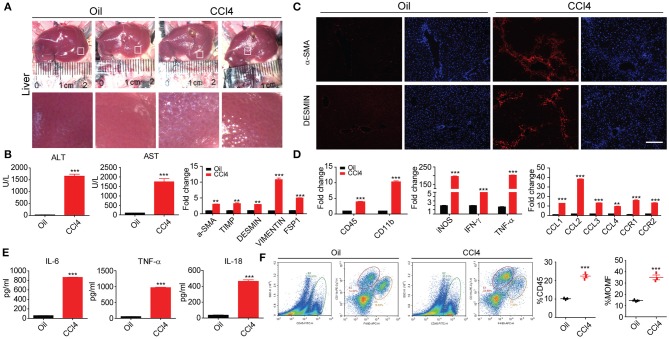
Inflammation is accompanied by liver fibrosis. **(A)** Macroscopic analyses of the murine liver fibrosis model in C57BL/6 mice by 8 week of two times per week i.p. CCl_4_ injection. Comparisons were made with control (injured oil) animals. **(B)** Histological characterization of hepatic fibrosis and myofibroblast activation by α-SMA and DESMIN immunohistochemistry. **(C)** Serum ALT and AST levels and other fibrosis protein levels (*n* = 10). **(D)** Whole-liver protein levels of Il-1β, Ccl2, Ccl3, and Cxcl2 measured by RT-PCR assay. **(E)** Supernatants were assayed for IL-6, TNF-α, and IL-18 levels by ELISA. **(F)** Flow cytometric analysis comparing expression of stated marker between inflammatory CD45+/CD11b^high^F4/80^mid^ hepatic macrophages. All data shown as mean ± SEM. *P < 0.05, **P < 0.01, ***P < 0.001.

CCl_4_-induced liver injury in mice is a most commonly used animal model of liver fibrosis that features hepatocyte injury and the activation of HSCs. In our study, 16 eight-week-old mice were randomly divided into two groups. The CCl_4_ group was conducted intraperitoneal injection of oil-dissolved CCl_4_ twice a week, and the oil group was set as control. We first used immunofluorescence, RT-PCR, and ELISA to characterize the pathological features. The macroscopic appearance of the liver revealed almost significant amount of collagen accumulation in the CCl_4_ treatment groups after 8 weeks, whereas the oil group was still normal (Figures 1A,B). Immunochemical staining exhibited that the α-SMA and DESMIN increased with liver fibrosis progression and other fibrosis-related genes were also remarkably enhanced (Figure 1C). Subsequently, the markers of liver injury in the serum were measured, along with aspartate aminotransferase (AST) and alanine aminotransferase (ALT) levels, given that AST and ALT are expected to abundantly distribute in injured hepatocytes and that the excessive release of these two enzymes into the serum can indicate the degree of hepatocyte injury. As illustrated in Figure 1C, AST and ALT levels increased dramatically, along with the notably up-regulated secretion and expression of inflammatory cytokines after CCl_4_ treatment (Figures 1D,E). In addition, our study also clarified its possible association with the aberrantly changed hepatic macrophage subsets. The total hepatic macrophages were assayed and detected as CD45^+^, CD11B^+^, and F4/80^+^ cells from the non-parenchymal cell (NPC) fraction after *in situ* perfusion of the hepatic portal vein and flow cytometry assay. Importantly, coinciding with fibrosis severity, liver resident macrophages, which often called Kupffer cells and detected as F4/80^high^ CD11B^intermediate^, were predominant in the control group (uninjured). Lowered proportion of resident macrophages was observed during the process of stimulated inflammation and fibrogenesis; CD11B^high^ F4/80^intermediate^ subset signifies a monocyte-derived recruited macrophage population that has increased progressively during fibrogenesis (Figure 1F). In summary, our data suggested that an initial cell injury can trigger inflammation to give rise to worsened liver fibrosis.

### miRNAs and Pathways That Are Correlated With Liver Fibrosis

**Figure 2:**
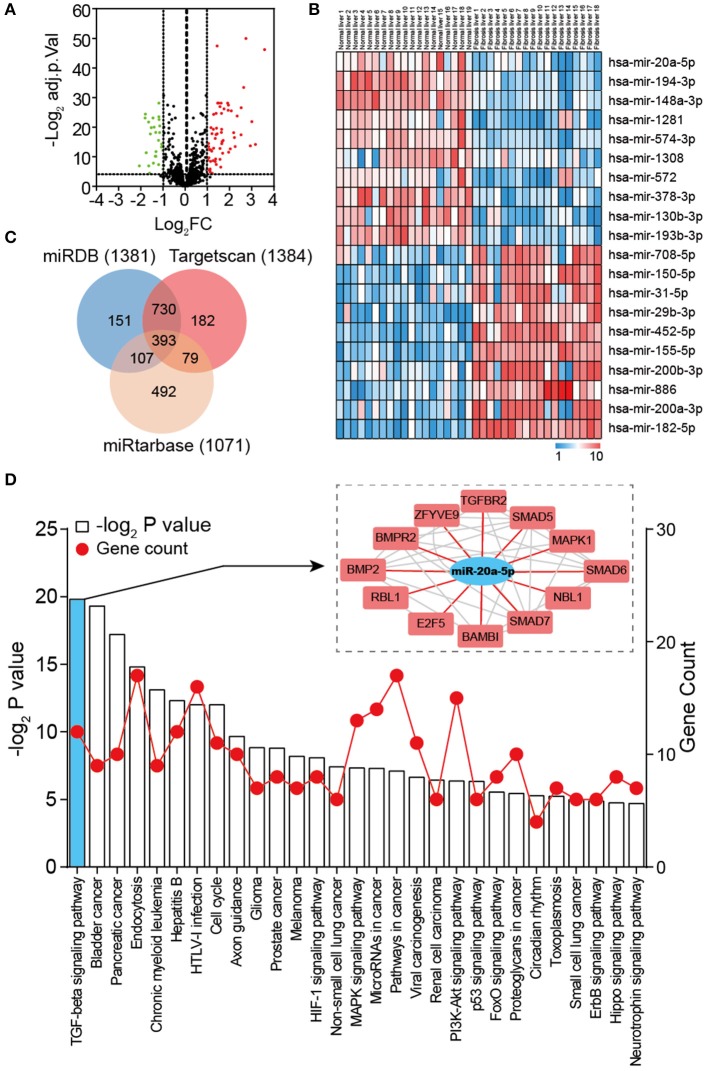
Global properties of miRNAs and pathways correlated with liver fibrosis. **(A)** Volcano plot of the DEMs (adjust *P* < 0.05 and |logFC| ≥ 2 were set as the cut-off criteria). **(B)** Heat map of the top 20 DEGs (top 10 up-regulated and 10 down-regulated genes). **(C)** Venn diagram of target genes predicted by miRDB, TargetScan, and miRtarbase. **(D)** KEGG pathway enrichment analysis of target genes of miR-20a-5p. The red lines represent gene count and the histogram represent –log_2_ (*P* value).

To identify DEMs of GSE40744 downloaded from GEO database, GEO2R tool was employed to perform the differential expression analysis following the protocol introduced in Materials and Methods section. Eighty nine miRNAs in total (62 up-regulated and 27 down-regulated) were ascertained to show significantly different expression in liver fibrosis biopsy specimen, reaching as high as two-fold aberration in comparison with normal ones (Figure 2A and Table S3). To ensure clearer visualization, the top 10 up-regulated and top 10 down-regulated miRNAs were selected as Figure 2B. As the most down-regulated miRNA, miR-20a-5p was picked for further analysis. 1381, 1384, and 1071 genes were detected as potential targets of miR-20a-5p through miRDB, TargetScan and miRtarbase, respectively. Three hundred and ninety three overlapping genes were identified as the targets of miR-20a-5p (Figure 2C and Table S4). Subsequently, enrichment analysis through KEGG database was carried out to identify the main pathways of these targets. Twenty nine significantly enriched KEGG pathways were identified (Figure 2D), including TGF-β signaling pathway, Bladder cancer, and Pancreatic cancer, et al. It was reported that TGF-β signaling pathway was of importance in liver fibrosis development (27). We hypothesized that miR-20a-5p played a part in the development of liver fibrosis by regulating TGF-β signaling pathway.

### miR-20a-5p Was Down-Regulated and Associated With Inflammation During Liver Fibrosis

**Figure 3:**
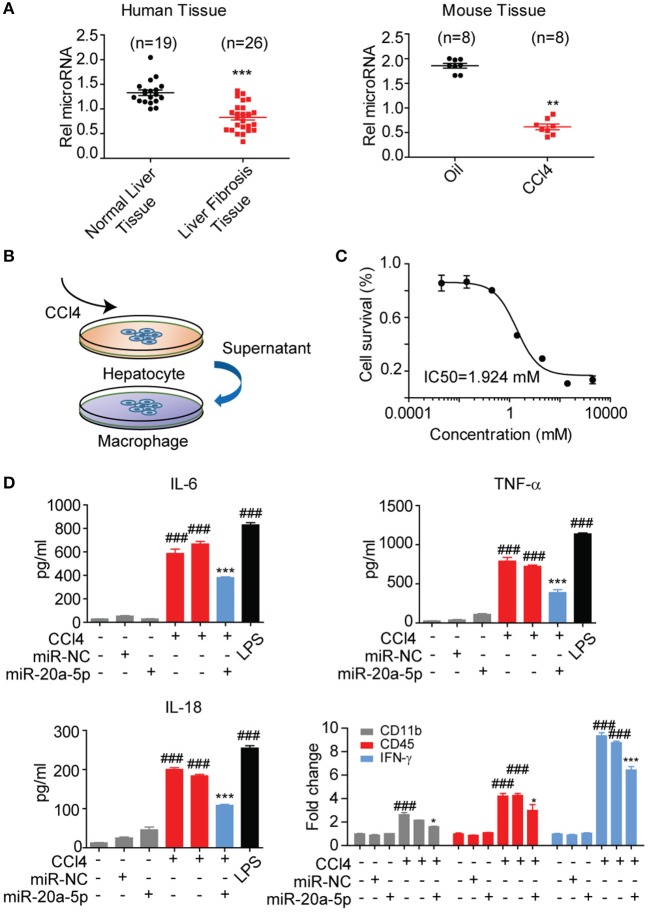
MiR-20a-5p was down-regulated and associated with inflammation during liver fibrosis. **(A)** miR-20a-5p expression in the clinical samples from normal (*n* = 10) or liver fibrosis patients (*n* = 20) were analyzed by qRT-PCR. **(B)** The schematic of the *in-vitro* cell model. **(C)** The concentration-response curves of CCl_4_ for hepatocyte injury model. **(D)** The cytokine levels of IL6, TNF-α, and IL-18 were determined in control cells and CCl4-cells transfected with miR-20a-5p or their respective NCs by ELISA and qRT-PCR. *p < 0.05, **p < 0.01, ***p < 0.001, ^*###*^*p* < 0.001, respectively. *Compared with CCl4 plus miR-NC and ^#^compared with the control.

To validate whether miR-20a-5p is a modulator in liver fibrosis, the expression level of miR-20a-5p was measured through qRT-PCR assay in liver tissues collected from patients, CCl_4_-induced mice model and healthy controls. In agreement with our assumption, miR-20a-5p expression level was significantly reduced in both tissue specimens of patients and CCl_4_-induced mice (Figure 3A). These results prompted us to further explore the function of miR-20a-5p in liver fibrosis. We built an *in-vitro* cell model to simulate the complex process of fibrosis (Figures 3B,C). Hepa1-6 cells were transfected with miR-20a-5p mimic followed by CCl_4_ treatment. Forty eight hours later, the culture supernatant was collected to treat Raw264.7 cells. ELISA assays showed that impaired-hepatocyte caused inflammation was blocked by restored miR-20a-5p, which was further confirmed by the other cytokines expression, e.g., CD11b, CD45, and INF-γ, the key markers widely accepted for inflammation test (Figure 3D, Figure S1). Our data indicate that miR-20a-5p expression is functionally related to inflammation during the onset and progression of liver fibrosis.

### miR-20a-5p Alleviated Liver Fibrosis Through TGF-β Signaling Pathway

After binding to its receptors, TGF-β1 can activate the transcription factor downstream the pathway, Smad 2 and Smad3, to mediate fibrosis, and the signaling is negatively mediated by Smad7.

**Figure 4:**
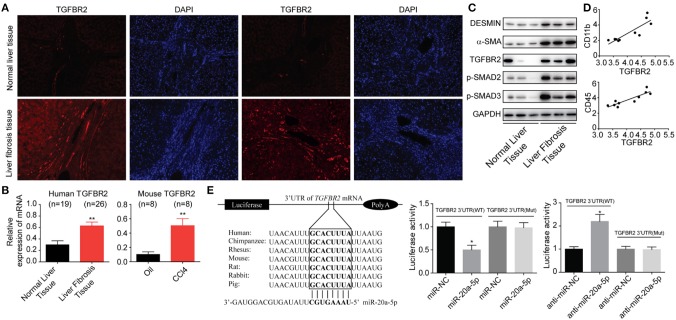
miR-20a-5p alleviated liver fibrosis through TGF-beta signaling pathway. **(A)** Immunohistochemical staining of TGFBR2 in human tissues from healthy controls and patients. **(B)** The protein levels of fibrosis markers and TGF-beta pathway makers. **(C)** The correlation between TGFBR2 and CD11b, CD45 mRNA expression level in liver fibrosis patients. **(D)** The TGFBR2 mRNA levels in human and mouse models. **(E)** The interaction between miR-20a-5p and TGFBR2 3′UTR. Luciferase activity was determined in cells co-transfected with different luciferase reporter vectors and miRNAs. All data shown as mean ± SEM. *P < 0.05, **P < 0.01, ***P < 0.001.

It is abundantly clear that TGF-β/Smad pathway is a major signal that activates HSCs and mediates fibrosis triggering downstream Smad 2 and Smad3 by TGF-β1. We have showed that TGFBR2 is one of the miR-20a-5p targets by searching the miRNA interactome dataset (Figure 2D). Thus, we initially investigated the expressions of TGFBR2 in liver fibrosis samples from patients. Immunofluorescence staining indicated that TGFBR2 expression level was notably enhanced in specimen collected from patients than that from healthy controls (Figures 4A,B). Analogously, the expression levels of both phosphorylated-Smad2 and phosphorylated-Smad3 were notably higher than those in normal tissues, suggesting the activation of TGF-β signaling pathway (Figure 4C). Because we demonstrated miR-20a-5p alleviated liver fibrosis may through lighten inflammation, we sought to evaluate the relevance between TGFBR2 and inflammation. As expected, the TGFBR2 expression exhibited significantly correlation with CD11b and CD45 (GSE80601, *r*^2^ = 0.9201 and 0.9786, respectively; both *P* < 0.0001) (Figure 4D). Finally, the 3′UTR sequence of TGFBR2 mRNA was cloned into the pGL3-Basic plasmid, in an attempt to ascertain the possible regulatory role of miR-20a-5p in the expression of TGFBR2 via binding to the predicted site. Our data demonstrated that miR-20a-5p mimics induced significantly inhibited luciferase activities of pGL3-TGFBR2 (WT), but no effect was observed on pGL3-TGFBR2 (Mut) (Figure 4E). Collectively, our data strongly suggests that miR-20a-5p down-regulation reinforce TGF-β signaling, at least in part, through alleviating to target TGFBR2 mRNA, leading to inflammation during liver fibrosis progression.

## Discussion

Aberrant hepatocyte death and persistent liver inflammation are recognized as drivers of liver fibrosis that in a chronic setting can promote HCC development (28). In the present study, we reported peripheral macrophage population accumulates during fibrosis. Besides, using microarray data of liver miRNomes, we measured the whole-genome miRNA expression of human liver fibrosis tissues and determined miR-20a-5p as a key modulate miRNA. The TaqMan probe-based qRT-PCR was performed to verify the predominance of miR-20a-5p through in both mouse and human samples. Furthermore, the present study demonstrated that lower level of miR-20a-5p exacerbates inflammation, whereas up-regulation of miR-20a-5p suppresses the releasing of cytokines.

Macrophages are “keystones” of liver architecture in both homeostasis and disease. Several studies have corroborated the central role played by macrophages in mediating inflammation and tissue fibrogenesis in several organ systems, but the progress is reverse (18, 29). Given the urgency and necessity to discover or develop an effective therapy for liver fibrosis, an increasing number of studies focus on analyzing miRNA mechanisms in fibrotic diseases, shedding light on the biological role of miR-21, miR-132, miR-155, miR-26a, and so forth. Previous studies demonstrated the elevated miR-155 expression in Kupffer cells after prolonged alcohol uptake, and that TNF served as a miR-155 target gene to give rise to liver inflammation (30, 31). miR-20a is one of miR-17/92 cluster members, which are located in the 13q31.1 region, which is largely involved in inflammatory. Overexpression of miR-20a could reduce the activity of inflammasome NLRP3 by mediating targeting thioredoxin-interacting protein (TXNIP) (32). Furthermore, miR-20a was reported to be beneficial to human aortic endothelial cells derived from Ox-LDL-induced inflammation through mediating TLR4 and TXNIP signaling (33). Moreover, miR-20a was also reported to regulate signal-regulatory protein α (SIRPα), resulting in macrophage infiltration, phagocytosis, and pro-inflammatory cytokine secretion (34). The exact part played by miR-20a-5p in the progression of liver fibrosis is yet to be elucidated. Herein, our data have shown that miR-20a-5p was distinctly decreased with advanced fibrosis and we develop a novel cell model to simulate the macrophage activation during fibrosis. Notably, restoration of miR-20a-5p suppresses inflammations caused by inhibited hepatocyte injury.

Since miR-20a-5p suppressed inflammation *in vitro*, exploring its underlying mechanism relevant to the disease process of fibrosis is necessary. We further observed the level of TGFBR2 up-regulated in patients compared to normal liver. In addition, miR-20a-5p could regulate TGFBR2 expression by directly binding to its 3′-UTR, while TGF-β pathway contributes to hepatotoxicity which influences macrophage activation. Among the multiple causative factors, it's well-known that TGF-β/Smad pathway is essential for liver fibrosis development (35, 36). Connection of TGF-β and its receptors, including TGFBR1 and TGFBR2 could endow it with the serine threonine kinase activity. TGF-β is always recognized as a pro-fibrogenic cytokine in TGF-β signaling pathway due to its function in HSC activation and ECM production (37–39). Recently, it has been revealed that TGF-β is essential for the development and critical features of multiple tissue-resident macrophages. What's more, TGF-β is required for the maintenance of expression pattern of the macrophage-specific homeostatic genes (40–42). Our data verified the contributions of TGF-β signaling pathway in hepatocytes to macrophage activity.

Together, our results highlight a critical function of miR-20a-5p in the liver fibrosis development, and provide the first evidence that miR-20a-5p maintains the survival of hepatocyte via TGF-β signaling pathway and that inhibits inflammation occur. Moreover, the reintroduction of miR-20a-5p enlightens a promising therapeutic strategy for the clinical intervention of liver fibrosis.

## Data Availability Statement

The datasets generated for this study can be found in the NCBI Gene Expression Omnibus (http://www.ncbi.nlm.nih.gov/geo/) (GSE40744).

## Ethics Statement

The studies involving human participants were reviewed and approved by Institutional Ethics Review Board of the Zhongshan Hospital. The patients/participants provided their written informed consent to participate in this study. The animal study was reviewed and approved by Institutional Ethics Review Board of the Zhongshan Hospital.

## Author Contributions

XF and QF design the concept, experimented and wrote the manuscript. JQ have done the system biology analysis. JC and YJ collated the data used in this project. ZD designed the problem, guided the study, and finalized the manuscript.

### Conflict of Interest

The authors declare that the research was conducted in the absence of any commercial or financial relationships that could be construed as a potential conflict of interest.

## Supplementary Material

Figure S1 Decreased miR-20a-5p reinforce inflammation during liver fibrosis progression. **(A)** The cytokine levels of IL6, TNF-α, and IL-18 were determined in control cells and CCl4-cells transfected with si-miR-20a-5p or their respective NCs by ELISA. Values are presented as mean ± SEM. ^*^*p* < 0.01 compared with CCl4 plus si-miR-NC and ^*###*^*p* < 0.001 compared with the control.

Table S1 Clinical characteristics of patients.

Table S2 List of primers for qTR-PCR.

Table S3 List of miRNA identified in miRNA profile.

Table S4 List of target genes regulated by miR-20a-5p.
