# Supplementary figures and images for: miR-20a-5p/TGFBR2 Axis Affects Pro-inflammatory Macrophages and Aggravates Liver Fibrosis
Source: Front Oncol. 2020 Feb 13;10:107. doi: 10.3389/fonc.2020.00107 (PMC7031347; doi:10.3389/fonc.2020.00107)

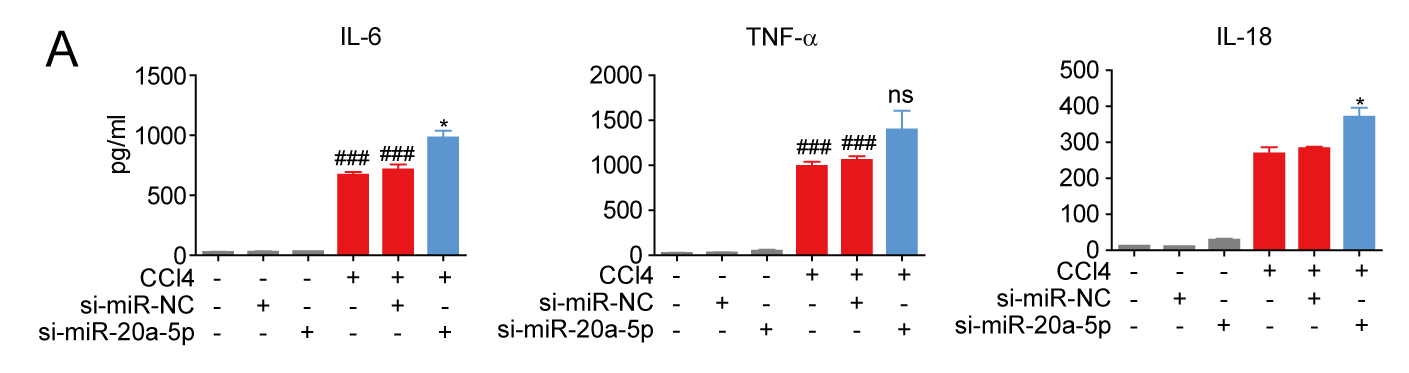

Supplement: Figure S1 — Decreased miR-20a-5p reinforce inflammation during liver fibrosis progression. (A) The cytokine levels of IL6, TNF-α, and IL-18 were determined in control cells and CCl4-cells transfected with si-miR-20a-5p or their respective NCs by ELISA. Values are presented as mean ± SEM. *p < 0.01 compared with CCl4 plus si-miR-NC and ###p < 0.001 compared with the control. [file Image_1.tif]
